# Aging erythrocyte membranes as biomimetic nanometer carriers of liver-targeting chromium poisoning treatment

**DOI:** 10.1080/10717544.2021.1949075

**Published:** 2021-07-08

**Authors:** Qing Yao, Guobao Yang, Hao Wang, Jingzhou Liu, Jinpeng Zheng, Bai Lv, Meiyan Yang, Yang Yang, Chunsheng Gao, Yongxue Guo

**Affiliations:** a School of Pharmaceutical Engineering, Shenyang Pharmaceutical University, Shenyang, PR China; b State Key Laboratory of Toxicology and Medical Countermeasures, Beijing Institute of Pharmacology and Toxicology, Beijing, PR China; c School of Pharmacy, Jiamusi University, Jiamusi, PR China; d School of Pharmacy, Qiqihar Medical University, Qiqihar, PR China

**Keywords:** Aging erythrocyte membranes, bionic nanocarrier, chromium poisoning, dimercaptosuccinic acid, liver-targeted

## Abstract

Chromium poisoning has become one of the most common heavy metal poisoning occupational diseases with high morbidity and mortality. However, most antidotes detoxify the whole body and are highly toxic. To achieve hepato-targeted chromium poisoning detoxification, a novel hepato-targeted strategy was developed using aging erythrocyte membranes (AEMs) as biomimetic material coated with a dimercaptosuccinic acid (DMSA) nanostructured lipid carrier to construct a biomimetic nano-drug delivery system. The particle size, potential, drug loading, encapsulation rate, *in vitro* release, and stability of the nanoparticles (NPs) were characterized. Confocal microscopy and flow cytometry showed that the prepared NPs could be phagocytized by RAW264.7 macrophage cells. The efficacy of AEM-DMSA-NPs for targeted liver detoxification was evaluated by *in vitro* MTT analysis and an *in vivo* model of chromium poisoning. The results showed that the NPs could safely and efficiently achieve targeted liver chromium poisoning detoxification. All the results indicated that the biomimetic nano-drug delivery system mediated by aging erythrocyte membranes and containing DMSA nanoparticles could be used as a novel therapeutic drug delivery system potentially targeting liver detoxification.

## 1. Introduction

Heavy metal poisoning, such as chromium poisoning, is an occupational disease that often occurs in industrial, agricultural, and mining activities. In recent years, chromium pollution in the environment and the threat to human and wildlife health have attracted great attention. As an important site of metabolism and detoxification in the body, the liver is one of the target organs of Cr(VI) toxicity. Exposure to Cr (VI), especially long-term exposure, can cause pathological changes and liver damage including hepatomegaly in the exposed population, with potential mutagenic and carcinogenic effects. The destruction of the liver structure will inevitably affect the metabolism of the liver cells and cause liver function changes. Then, many enzymes and proteins in the liver cells will enter the blood, resulting in abnormal enzymatic activity and changes in the protein content in the blood (Amin et al., 2017; Wang and Zeng, 2019). At present, the common chromium antidotes are mainly dimercaptopropanol, dimercaptosuccinic acid, penicillamine, and EDTA, but these drugs are mainly used for systemic detoxification and have certain toxic and side effects, but these agents cannot achieve targeted detoxification of the liver (Chisolm, 1992). Therefore, liver-targeted chromium poisoning detoxification is a challenging undertaking.

In recent years, liver-targeting therapy has been applied in the clinical treatment of liver disease. Wang et al. used liver sinusoidal endothelial cells (LSECs) to target nonalcoholic fatty liver disease (NAFLD), which effectively prevented the occurrence of inflammation and is an attractive treatment strategy for NAFLD. However, this strategy involves a variety of changes in the morphology and function of the LSECs, leading to low clinical efficacy and poor stability *in vivo* (Wang & Peng, 2021). Yang et al. proposed a new strategy of drug delivery using extracellular vesicles (EVs) targeting liver inflammation as a carrier. This strategy could effectively treat liver diseases, but due to the poor biological effect of a single EV, most drugs have disadvantages such as poor selectivity of the target organs, and can even induce liver injury (Yang and Liu, 2020). Therefore, the selection of the appropriate drug delivery system to improve the selectivity and targeting of the current clinical drugs is considered to be an effective measure to improve the efficacy of liver disease treatments and reduce adverse reactions (Xia et al., 2021). Bionic nano-drug delivery systems are considered to be the best new candidate drug for liver disease treatment due to their characteristics of target site accumulation, good biocompatibility, good pharmacological activity, and nontoxic side effects. Bionic drug delivery systems are novel drug delivery systems developed by directly utilizing or imitating complex biological structures and processes (Fang et al., 2018). The commonly used biomimetic materials include cell membranes, lipoproteins, and exosomes. Red blood cells (RBCs) are the most readily available and abundant cells in the body. Young red blood cells express antigens not seen as foreign by macrophages. The expression of CD47 on aged red blood cells is downregulated, causing their eventual phagocytosis by macrophages and accumulation in the liver. Because of the natural characteristics of aging RBCs, aging RBCs can be used as carriers to deliver drugs to achieve the purpose of liver-targeted therapy. But cells suitable for insoluble drugs are limited because of drug loading capacity. To effectively solve this problem, many studies have focused on drug-loaded nanoparticles. Among them, nanostructured lipid carriers (NLC) are a widely used biological material due to higher drug-loading capacity, lower drug-coating leakage rate, good biocompatibility, and other characteristics, becoming the first choice of carrier systems (Chen et al., 2008; Yuan et al., 2013). Therefore, we planned to use aging erythrocyte membranes as biomimetic material, encapsulate nanoparticles loaded with drugs, and accumulate them in the liver by macrophage phagocytosis to detoxify liver chromium poisoning.

Dimercaptosuccinic acid (DMSA) is a new antidote developed in China. Compared to other antidotes, DMSA has many advantages, such as low toxicity, high bioavailability, and strong patient compliance, making it one of the first choices of heavy metal detoxification drugs (Graziano, 1986). After DMSA is administered, two active sulfhydryl groups in the molecule can seize the metals bound to the enzyme system in the tissue to form a stable water-soluble chelate, which is excreted in the urine, and enzymes are free and restore their function, thus, relieving the poisoning symptoms caused by heavy metals (Chen et al., 2014; Ma & Gong, 2014). However, the by-products produced by DMSA after entering the body cause more serious gastrointestinal reactions and hypercomplexation syndrome, which will affect the therapeutic effect and health of the patients. The long-term or one-time use of large doses of DMSA causes damage to liver and kidney function, including irreversible damage. To effectively reduce the damage caused by DMSA to the human liver and kidneys, we considered loading DMSA on nanoparticles and prepare them as biomimetic nanocarriers to achieve a slow-release, reduce the side effects of the drugs in the human body, and improve the bioavailability of the drugs.

We planned to achieve the precise delivery of drugs through a ‘system targeting’ strategy. First, a bionic drug delivery system was constructed by wrapping aging erythrocyte membranes damaged by hydrogen peroxide oxidation with a nanostructured lipid carrier loaded with DMSA. Then, the biomimetic nanocarriers were injected into mice with chromium poisoning by tail vein injection, and the biomimetic nanocarriers were engulfed by macrophages, transported, and accumulated in the liver. DMSA played a role in facilitating biomaterials to achieve liver-targeted chromium poisoning detoxification. Targeted liver detoxification has important research value and social significance in treating chromium poisoning and alleviating the liver toxicity and liver injury caused by heavy metal chromium poisoning.

## 2. Materials and methods

### 2.1. Materials

Dimercaptosuccinic acid (DMSA) was purchased from Sigma-Aldrich (Beijing, China). All chemicals were of reagent grade and obtained from Sinopharm Chemical Reagent Co. Ltd. (Mainland, China) unless otherwise stated.

RAW264.7 cells were purchased from Punosel Life Sciences (Wuhan, China). RAW264.7 cells were maintained in a culture medium consisting of Dulbecco’s modified Eagle’s medium (DMEM) (Shanghai, China) supplemented with 10% fetal bovine serum (FBS) and 1% penicillin–streptomycin. The cells were maintained in a 37 °C humidified incubator in a 5% CO_2_ atmosphere.

Both Kunming mice (20–25 g) and Sprague–Dawley rats (190–210 g) were purchased from Spyfe Biotech (Beijing, China). All animal experiments complied with the code of ethics in research, and the training and testing of drugs issued by the Animal Care and Use Ethics Committee of the Beijing Institute of Pharmacology and Toxicology.

### 2.2. The preparation of nanoparticles

The DMSA-NLC nanoparticles were prepared by melting and ultrasonication. In a 65 °C water bath, glycerol monostearate (45 mg) was dissolved in 1 mL of methanol and acetone (1:1), followed by triglyceride chain (45 μL) and sulfhydryl succinic acid (1.40 mg), and the mixed solution was used as the oil phase. Sodium deoxycholate (23 mg) was dissolved in 20 mL of distilled water as the aqueous phase in a 65 °C water bath. The oil phase was blended by dropwise addition to the water phase and stirring for 1 h. The final solution was ultrasonically mixed for 5 min (500 W) in an ice-water bath and then filtered through 0.45 μm and 0.22 μm membranes successively to obtain the DMSA-NLC suspension (Chen & Gu, 2014).

### 2.3. Characterization of the nanoparticles

The mean diameter and particle distribution of DMSA-NLC were measured by dynamic light scattering (DLS) (Litesizer 500, Anton Parr, Austria). The morphology of streptavidin-NPs was characterized by transmission electron microscopy (TEM) (HITACHI, H-7650, Tokyo, Japan).

### 2.4. Preparation of aged red blood cells

Aging red blood cells were prepared by hydrogen peroxide oxidation damage. Erythrocyte suspension (2 mL of 6%) was added to each well of a 6-well plate and divided into six groups, including the control group and the H_10_, H_50_, H_100_, H_200_, and H_400_ groups (triplicate) and incubated in a 37 °C and 5% CO_2_ incubator for 12 h. Flow cytometry separation was performed to detect the aging of red blood cells induced by hydrogen peroxide, and choose the best concentration of red blood cell aging.

### 2.5. Preparation of aging erythrocyte membranes

The low-permeability method was used to extract aging erythrocyte membranes (AEMs). The aging red blood cell suspension was centrifuged for 10 min (4 °C, 3000 r/min), the supernatant was removed and replaced with the same volume of 4 °C saline to disperse the precipitate. The process was repeated three times, and the last wash was with 5 volumes of 0.25% low-permeability physiological saline, the pellet was dispersed and incubated for 30 min in a 4 °C refrigerator. The material was centrifuged for 10 min (4 °C, 3000 r/min), and discard the supernatant. Five volumes of 0.25% low-permeability saline were used to wash the pellet three times. After the final centrifugation, the pale pink lumps of precipitate are the aged erythrocyte membrane.

### 2.6. The preparation of AEM-DMSA-NPs

FITC-labeled NLC were prepared by incubation method. Specifically, FITC (1 mg) and NLC suspension (10 mL) were incubated for 12 h. The aging erythrocyte membranes were mixed with FITC-labeled NLC at the ratios of 1:1, 1:2, 1:5, 1:8, and 1:10 (v/v). The combination efficiency of NLC and the aging erythrocyte membranes was determined by flow cytometry after ultrasonic incubation at 300 W and 59 Hz for 5 min.

The aging red cell membranes and nanometer carrier mixed 1:5 (v/v) were ultrasonicated at 300 W and 59 Hz for 5 min, then extruded through miniature extrusion membranes of 400 nm, 200 nm, and 100 nm, squeezing each particle size more than 30 times back and forth. In this study, AEM-DMSA-NPs containing dimercaptobutadiic acid wrapped in aging erythrocyte membranes with uniform particle size were obtained and stored refrigerated at 4 °C.

### 2.7. Drug-loading and encapsulation efficiency

The drug-loading (DL) and encapsulation efficiency (EE) of micelles for DMSA were calculated by titration. The nanosuspension (400 μL) was placed in an ultra-speed centrifuge tube and centrifuged at 10,000 rpm/min for 20 min to determine the free DMSA content in the filtrate. The suspension (500 μL) was added to a 10 mL flask, with a fixed volume of acetonitrile and ultrasonicated to determine the total amount of DMSA. DMSA in the sample was measured using the content determination method described above. The measurements were performed in triplicate.

### 2.8. *In vitro* release and stability study

The release of DMSA-NLC and AEM-DMSA-NPs *in vitro* was analyzed by the dialysis method. DMSA-NLC and AEM-DMSA-NPs nanosuspensions (2 mL) were added to a dialysis bag (molecular weight cutoff: 3500 Da) and directly immersed into phosphate-buffered saline (PBS) (0.1 M, pH 7.4, 50 mL) at 37 °C. Samples (5 mL) were extracted at preset time points, and an equal volume of PBS was added. DMSA in the sample was measured using the content determination method described above.

For the stability study, AEM-DMSA-NP nanoparticle suspension liquid was diluted 30 times with PBS and FBS and put in a stability analyzer at 37 °C, scanned at the preset time, and the stability of the sample was determined by back scattering (BS) and the transmission (T) curve with the change of time.

### 2.9. Cell culture

Macrophage RAW264.7 cell cultures were maintained at 37 °C in a 5% CO_2_ incubator, in DMEM medium (containing 10% FBS and 1% penicillin/streptomycin solution (P/S)), with a change of medium every 2–3 days (Yu et al., 2016; Li et al., 2018).

### 2.10. Cell uptake

RAW264.7 cells were inoculated into 6-well plates at 1 × 10^6^ cells/mL and incubated for 24 h. Then, nanoparticles modified with Cou 6 or Cou 6-H_2_O_2_ were added to each well and incubated for 2 h. The cells were fixed in 4% paraformaldehyde (2 mL per well for 15 min), then Hoechst 33258 (10 μg/mL, 2 mL) was added to stain the nuclei for 20 min (Hu et al., 2016). Finally, the cells were washed with PBS and observed by confocal laser microscopy.

For the flow cytometry separation study, RAW264.7 cells were incubated and cultured similarly, except for the addition of Hoechst 33258.

### 2.11. Cytotoxicity

To measure the cellular toxicity of DMSA, RAW264.7 cells were seeded into 96-well plates (1 × 10^6^/well) in DMEM at 37 °C in a 5% CO_2_ atmosphere. After incubation for 24 h, the cells were treated with DMSA, DMSA-NLC, or AEM-DMSA-NPs and incubated for another 24 h (Wang et al., 2010). After incubation, the un-phagocytosed free DMSA was discarded. The culture medium (100 μL) and MTT (100 μL) were co-cultured for 4 h, then dimethyl sulfoxide (DMSO, 150 μL) was added, and the absorbance was measured at 570 nm with a microplate analyzer after shaking for 10 min (Zhuang et al., 2010; Wang & Chu, 2013; Li et al., 2014).

### 2.12. *In vivo* safety evaluation

Healthy rats were randomly divided into four groups (*n* = 3) and treated with saline (control), DMSA-NLC, and AEM-DMSA-NPs via tail vein injection. Bodyweight was recorded every other day. On day 14, the rats were euthanized, and samples of the heart, liver, spleen, lung, and kidney were collected and routinely stained by hematoxylin and eosin (HE).

### 2.13. *In vivo* imaging

KM mice were injected with 200 μL of normal saline, DiR-free-DMSA, DiR-DMSA-NLC, and DiR-AEM-DMSA-NPs (*n* = 3) into the tail vein. After 2, 4, 6, 8, 12, 24, 36, and 48 h, the mice were anesthetized with 3% isoflurane, and *in vivo* imaging was performed. The fluorescence signal of DiR in mice was quantified using the Living Image software (SAS Inc., Cary, NC). After *in vivo* imaging, the mice were euthanized, and tissue samples of the heart, liver, spleen, lung, kidney, and brain were removed for imaging.

### 2.14. Potassium dichromate modeling

Male mice were randomly divided into four groups (*n* = 3) and injected with saline (containing 1, 5, and 10 µg/mL potassium dichromate) through the tail vein. Bodyweight was recorded the next day. After 15 days, the mice were euthanized, and all organs and blood were collected for chromium content determination and biochemical analysis.

### 2.15. Pharmacodynamic study

Male mice were randomly assigned (*n* = 3) and injected with saline, potassium dichromate (30 mg/kg), free-DMSA, DMSA-NLC, and AEM-DMSA-NPs (1, 5, and 10 mg/kg) through the tail vein. First, a mouse model of chromium poisoning was established with a dose of 30 mg/kg potassium dichromate. Doses of 5 mg/kg (based on DMSA content) were given every other day for a total of four doses starting on day 8 after modeling. The mice were weighed before each administration, the data were recorded, and a weight-time change chart was generated.

### 2.16. Tissue distribution

KM mice were randomly assigned (*n* = 3) and injected with normal saline (0.5 h before administration), DiR-labeled free-DMSA, DMSA-NLC, and AEM-DMSA-NPs through the tail vein at 5 mg/kg. The eyeball blood was collected 0.5 h before injection and 0.5, 2, 4, 6, 10, 18, 24, 36, 48, and 72 h after injection. After dislocation of the cervical spine, tissue samples of the heart, liver, spleen, lung, kidney, and brain were taken and put into a homogenizer. Two times the weight of saline was added, and the material was homogenized. The tissue homogenate, plasma (200 µL), and three times the amount of precipitant (methanol:HCl 96:4) were mixed evenly. Centrifugation was carried out at 8000 rpm/min for 10 min by an eddy vortex for 30 s. The supernatant was removed to measure the fluorescence intensity by a microplate analyzer.

### 2.17. Pharmacokinetic study

SD rats were randomly divided into four groups (*n* = 3), fasted without water for 12 h before the experiment, and blood was taken from the fundus venous plexus for controls. Saline, free-DMSA, DMSA-NLC, and AEM-DMSA-NP suspensions were injected at a dose of 10 mg/kg (measured by the DMSA content). At 0.17, 0.5, 0.75, 1, 2, 4, 6, 8, 10, 12, 16, 20, 24, and 48 h after administration, 500 µL of blood was collected from the fundus venous plexus of rats and placed in a centrifuge tube (containing heparin sodium) and swirled for 1 min (Liu et al., 2019). Centrifugation was carried out at 10,000 rpm/min at 4 °C for 10 min. Plasma was separated and the DMSA content in plasma was determined by fluorescence spectrophotometry (Shi et al., 2013; Wang et al., 2017).

### 2.18. Statistical analysis

All data are presented as the means and standard deviation (SD). The data were analyzed using one-way ANOVA coupled with Dunnett’s post hoc analysis for multigroup comparisons (Chu et al., 2020). Comparison between two groups was accomplished via an unpaired two-tailed student’s *t*-test. The probability of survival was estimated by the Kaplan–Meier method and compared by the log-rank test (Ying & Zong, 2002). The relative fluorescence and immunohistochemistry staining intensity of at least three samples per group with three different sections per sample were assessed using ImageJ software (NIH Image J system, Bethesda, MD). *p* < .05 indicated a statistically significant difference.

## 3. Results and discussion

### 3.1. Preparation and characterization of AEM-DMSA-NPs

**Figure 1.**
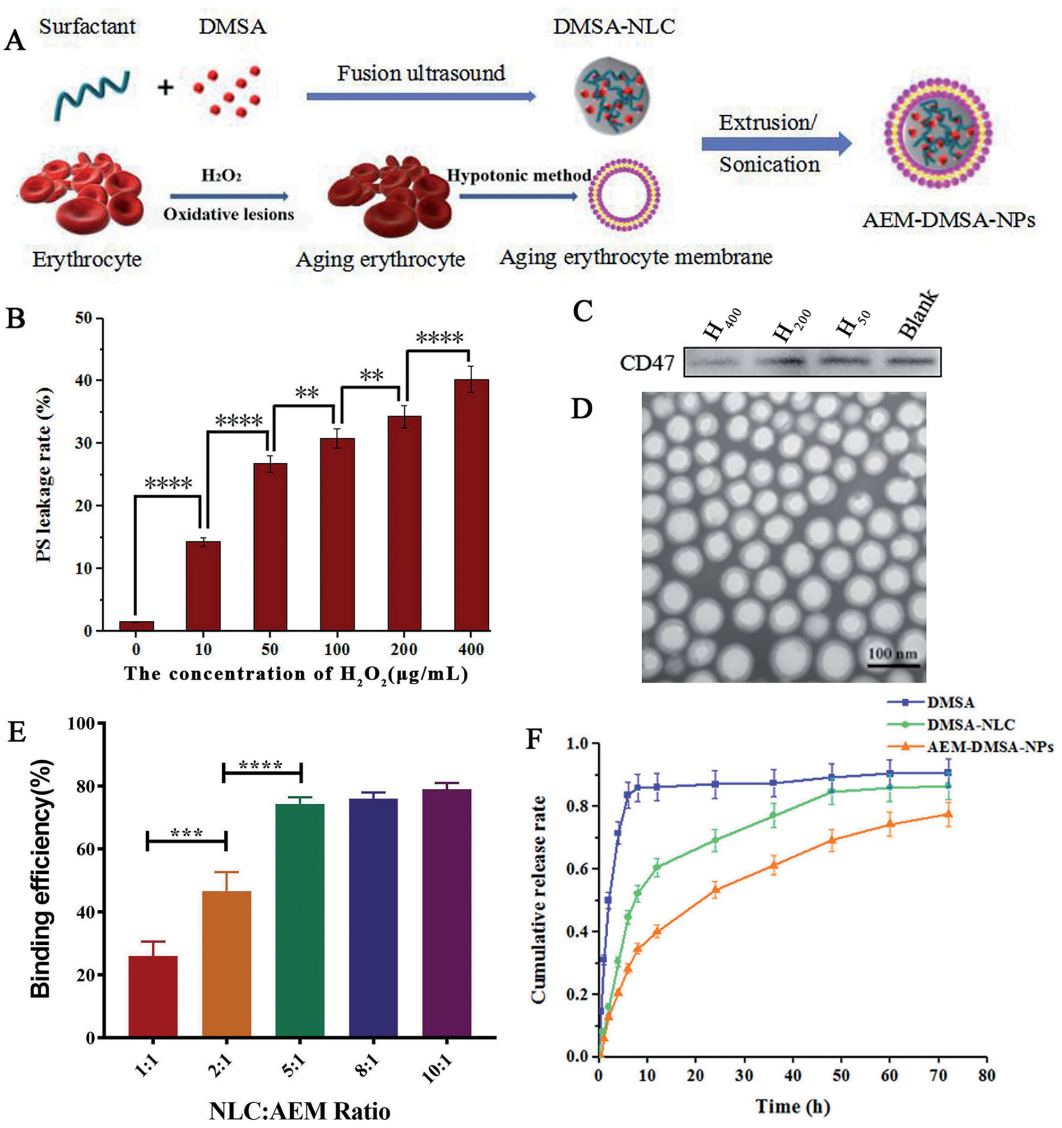
Preparation and characterization of NPs. (A) Schematic of the preparation of NPs. (B) The PS leakage rate of erythrocytes damaged by H_2_O_2_ oxidation. The data are presented as the means ± SD (*n* = 3). *indicates *p* < .05. (C) CD47 protein expression. (D) TEM image of the NPs. (E) Nanoparticle binding efficiency. The data are presented as the means ± SD (*n* = 3). *indicates *p* < .05. (F) *In vitro* DMSA release from NLC and NPs in PBS (pH 7.4) at 37 °C. The data are presented as means ± SD (*n* = 3).

The AEM-DMSA-NP preparation process is shown in Figure 1(A). First, the nanostructured lipid carriers loaded with DMSA were prepared by the melting ultrasonic method. Then, the red blood cells were aged by oxidative damage, and the cell membranes of the aged red blood cells were extracted. AEM-DMSA-NPs were prepared by liposome extrusion. After packaging in DMSA, the particle size of DMSA-NLC was 121.89 ± 2.36 nm, and the poly-dispersion coefficient was 0.11 ± 0.04, indicating that it had a narrow distribution. The zeta potential of NLC was 52.43 ± 3.56 mV, indicating that NLC was highly stable. In addition, the TEM image indicated that the particle size was similar to that measured by the laser particle size analyzer. The encapsulation efficiency (EE) and drug-loading (DL) of DMSA-NLC were relatively high (EE, 75.93 ± 0.54; DL, 2.58 ± 0.05).

Hydrogen peroxide was used to produce the oxidative damage of red blood cells. To determine the optimal amount of hydrogen peroxide, different concentrations of a fixed volume of hydrogen peroxide were used to treat the red blood cells. As shown in Figure 1(B), the flow cytometry measurements of extracellular phosphatidylserine (PS) exposure showed that when the concentration of H_2_O_2_ was increased from 10 µg/mL to 400 µg/mL, the level of PS exposure in the red blood cells was significantly increased. The expression of CD47 protein on the cells was detected by the Western blot method, and the results (Figure 1(C)) showed almost no expression at CD47 at a concentration of 400 µg/mL. Therefore, H_2_O_2_ at a concentration of 400 µg/mL was selected for the oxidative damage of red blood cells.

After producing oxidative damage, we evaluated whether the aging erythrocyte membranes could effectively encapsulate DMSA-NLC. For this, we first visualized the assembly of DMSA-NLC on the aging erythrocyte membranes by TEM, and the results are shown in Figure 1(D). AEM-DMSA-NPs had an obvious core–shell structure. Then, we quantitatively evaluated the combination of AEM and DMSA-NLC. FITC-labeled aging erythrocyte membranes were incubated with FITC-labeled nanoparticles in the range of 1:1 to 10:1, and the binding efficiency of NLC to the aging erythrocyte membranes was evaluated by flow cytometry (FCM) by measuring FITC fluorescence. As shown in Figure 1(E), the FCM results confirmed the effective assembly of NLC on the aging erythrocyte membranes. The binding efficiency of NLC and AEM continued to increase. When the nanoparticle/cell ratio increased to 8:1/10:1, the binding efficiency of NLC did not show a significant enhancement, which may be attributed to the saturation degree of the aged red blood cells. Therefore, an NLC/AEM ratio of 5:1 was chosen for the subsequent studies.

The purpose of the *in vitro* release study was to explore the drug release characteristics of the biomimetic nanocarriers combined with AEM and DMSA-NLC. The drug release of the free-DMSA, DMSA-NLC, and AEM-DMSA-NPs was investigated by the dialysis method. As shown in Figure 1(F), free-DMSA showed an obvious burst-release phenomenon. However, the drug release behavior of AEM-DMSA-NPs was similar to that of DMSA-NLC, both of which were continuously and slowly released, indicating that the aging erythrocytic membrane binding had limited influence on the physical and chemical properties of the nanosystem. Therefore, the slow-release nature will help prevent rapid leakage of the drug as it passes through the body, and increase drug accumulation at the target site.

To evaluate the stability of the AEM-DMSA-NPs in FBS, the particle size and the potential of the AEM-DMSA-NPs were measured by a laser particle size analyzer within 72 h. The results indicated that the aging erythrocyte membranes still had strong stability after combining with NLC loaded with DMSA.

### 3.2. *In vitro* cytotoxicity study and cell absorption

**Figure 2.**
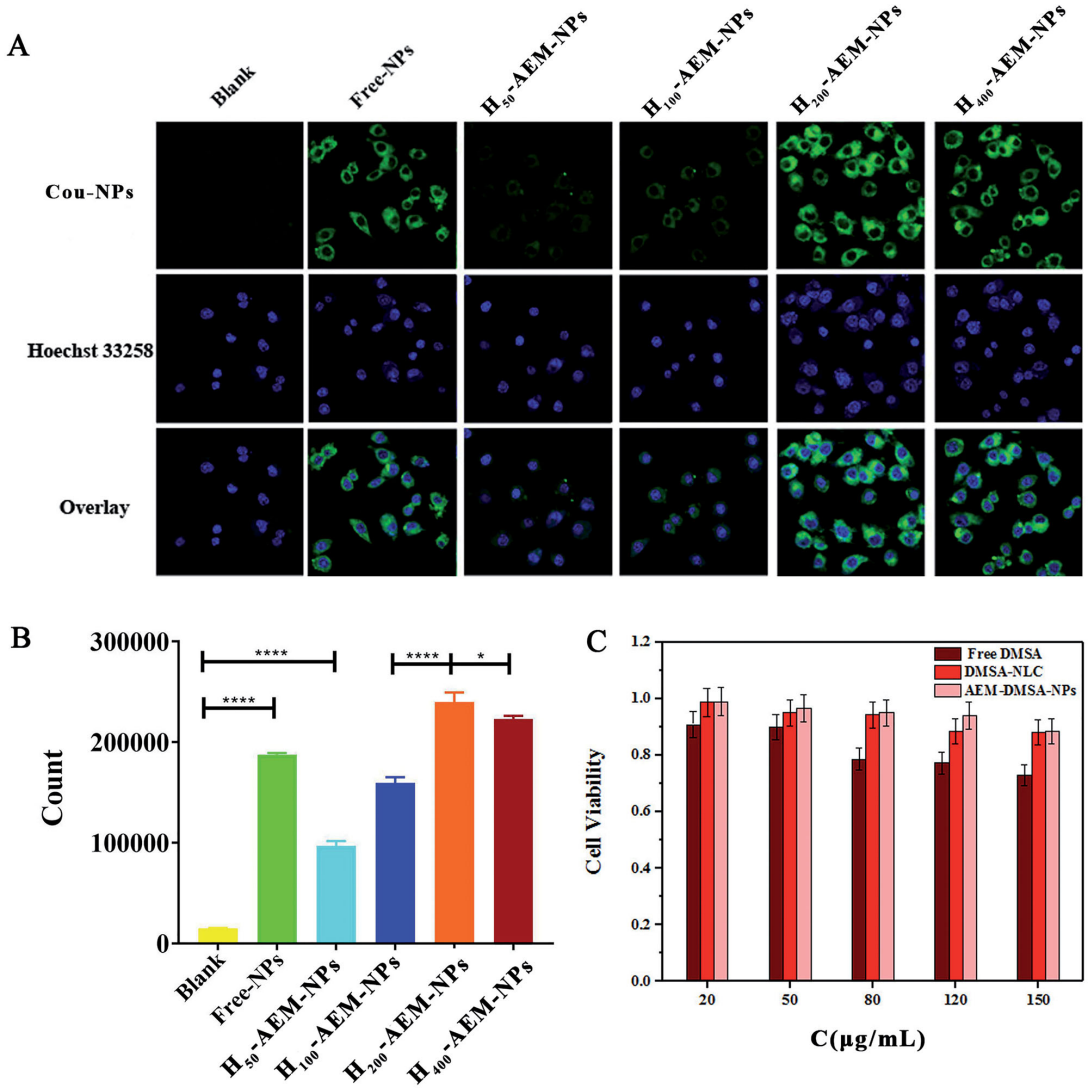
Cell uptake and cytotoxicity of NPs. (A) After RAW264.7 cells were incubated with coumarin-6 loaded NPs for 2 h and then stained with 4% paraformaldehyde, fluorescent images were observed by confocal microscopy. Scale bar: 50 μm. (B) The fluorescent images were observed using FCM. The data are presented as the means ± SD (*n* = 3). *indicates *p* < .05. (C) Cell viability of RAW264.7 cells incubated for 72 h with different concentrations of free-DMSA, DMSA-NLC, and AEM-DMSA-NPs. The data are presented as the means ± SD (*n* = 3).

In addition to having the right physiochemical properties, the ideal drug delivery system should have minimal toxicity and good absorption. To study the phagocytosis efficiency of macrophages to phagocytose AEM-DMSA-NPs, laser confocal and FCM were used to detect the phagocytosis ratio of RAW264.7 cells to serum-pretreated AEM-DMSA-NPs at 24 h. As shown in Figure 2(A), RAW264.7 cells had a certain uptake of Cou6-labeled DMSA-NLC. The uptake effect of NPs wrapped in erythrocyte membranes damaged by H_2_O_2_ oxidation increased with increases in concentration. FCM showed (Figure 2(B)) that RAW264.7 cells had a certain absorptive capacity for both DMSA-NLC and NPs compared to the blank group. However, there were also significant differences between the different concentrations of H_2_O_2_-modified AEM-DMSA-NPs in each group. When the concentration of H_2_O_2_ was 200 µg/mL, RAW264.7 cells had the strongest NP absorption capacity.

The cytotoxicity of AEM-DMSA-NPs as a carrier loaded with DMSA to macrophages is a key problem in cell-mediated simulated biological delivery systems. Cytotoxicity was verified in a time-varying MTT assay. As shown in Figure 2(C), after 24 h, free-DMSA showed cytotoxicity to macrophages in a concentration-dependent manner, and the cell survival rate was 72.9% at 150 µg/mL. DMSA-NLC and AEM-DMSA-NPs had almost no effects on the cells, and the survival rate of the cells cultured with different concentrations of NPs was 90%. In addition, AEM-DMSA-NPs showed reduced cytotoxicity compared to DMSA at the same concentration. Thus, this reduced cytotoxicity was critical for the detoxification of chromium, and the designed bionic nano-drug delivery system could be absorbed by RAW264.7 cells and delivered to the liver for drug release.

### 3.3. *In vivo* safety and targeting studies

**Figure 3.**
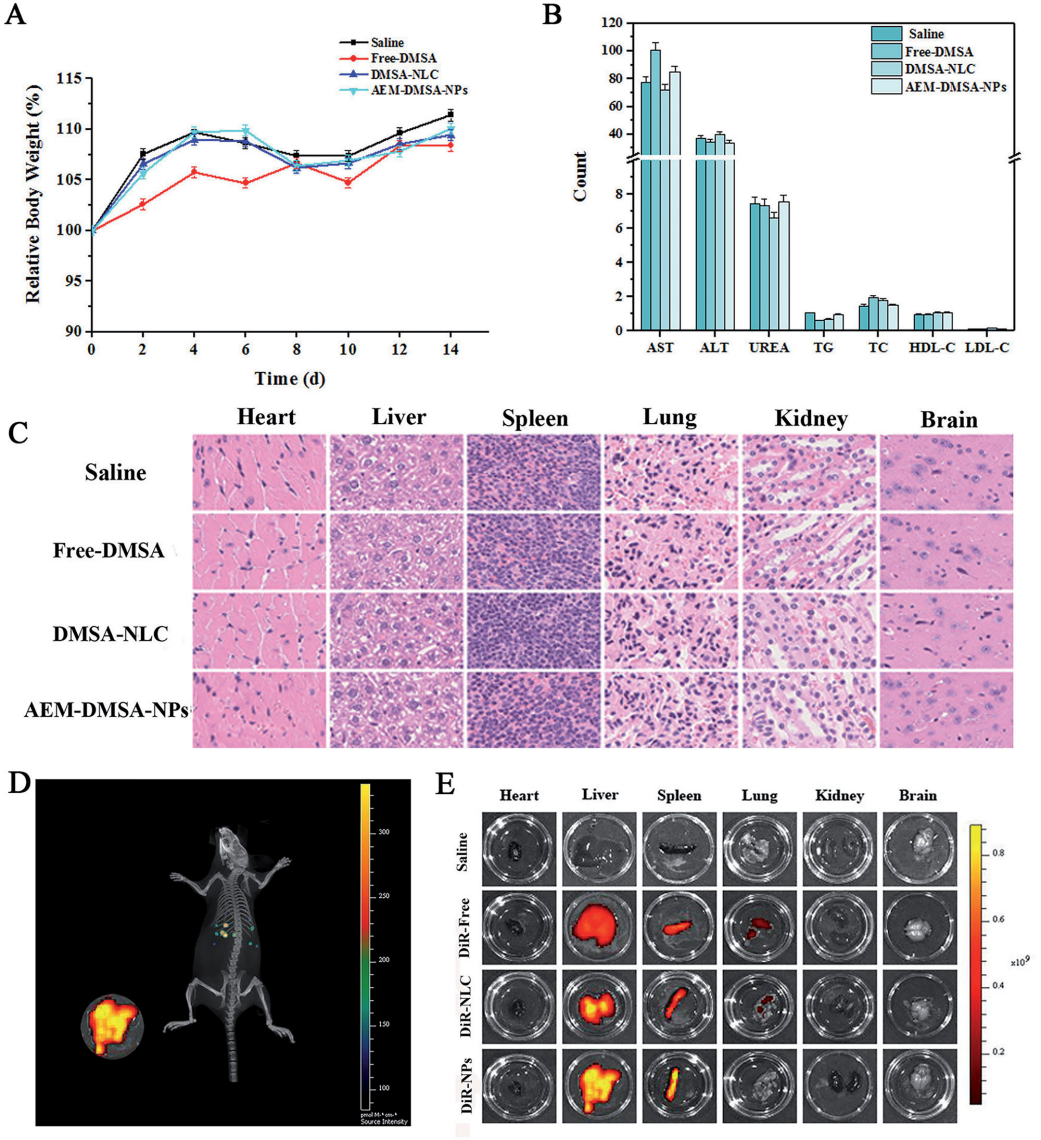
*In vivo* safety and targeting test. (A) Bodyweight changes. The data are presented as the means ± SD (*n* = 3). (B) Changes in biochemical indexes. (C) HE staining of major organ sections after various treatments. (D) *In vivo* real-time imaging of different DiR-tagged samples in the liver showing the biodistribution of nanocarriers in mice tissues. (E) The distribution of different drugs in the heart, liver, spleen, lung, kidney, and brain.

For *in vivo* safety evaluation, the relative rate of body weight change of the mice was determined, as shown in Figure 3(A). There was no significant difference in body weight in the study mice over 14 days. In addition, on the 15th day, the blood was collected from healthy mice for routine blood measurement, and the results are shown in Figure 3(B). There were no significant differences in biochemical indexes between the groups. HE staining can clearly present the pathological changes of various animal tissues and organs. On the 15th day, tissue sections of the heart, liver, spleen, lung, kidney, and brain were analyzed by HE staining. As shown in Figure 3(C), no indicators of damage to these organs were observed after treatment compared to the saline (control) group, indicating that intravenous AEM-DMSA-NPs did not cause systemic toxicity. Taken together, these results confirmed the low toxicity and good biocompatibility of NLC nanoparticles, which may be due to the biodegradation of NLC nanoparticles and the endogenous properties of aging red blood cells.

The DiR-labeled vector was injected intravenously into healthy mice, and the distribution of the vector in mice could be observed intuitively by *in vivo* imaging. Based on whole-body imaging (Figure 3(D)), DiR-labeled nanoparticles had a strong ability to target the liver. After 12 h, the mice were euthanized, and the main tissues and organs were dissected. *In vitro* imaging analysis was conducted under the same conditions, and the results are shown in Figure 3(E). There was almost no fluorescence signal in the normal saline group, which proved that the fluorescence emitted by the mice and normal saline did not significantly interfere with the experiment. In the treatment group, there was more distribution in the liver and spleen, and the fluorescence signal in the DiR-AEM-DMSA-NP group was very strong in the liver. It is also strongly distributed in the spleen. This further indicated that the AEM-DMSA-NPs had liver detoxifying targeting ability.

### 3.4. Chromium poisoning detoxifying ability of AEM-DMSA-NPs

**Figure 4.**
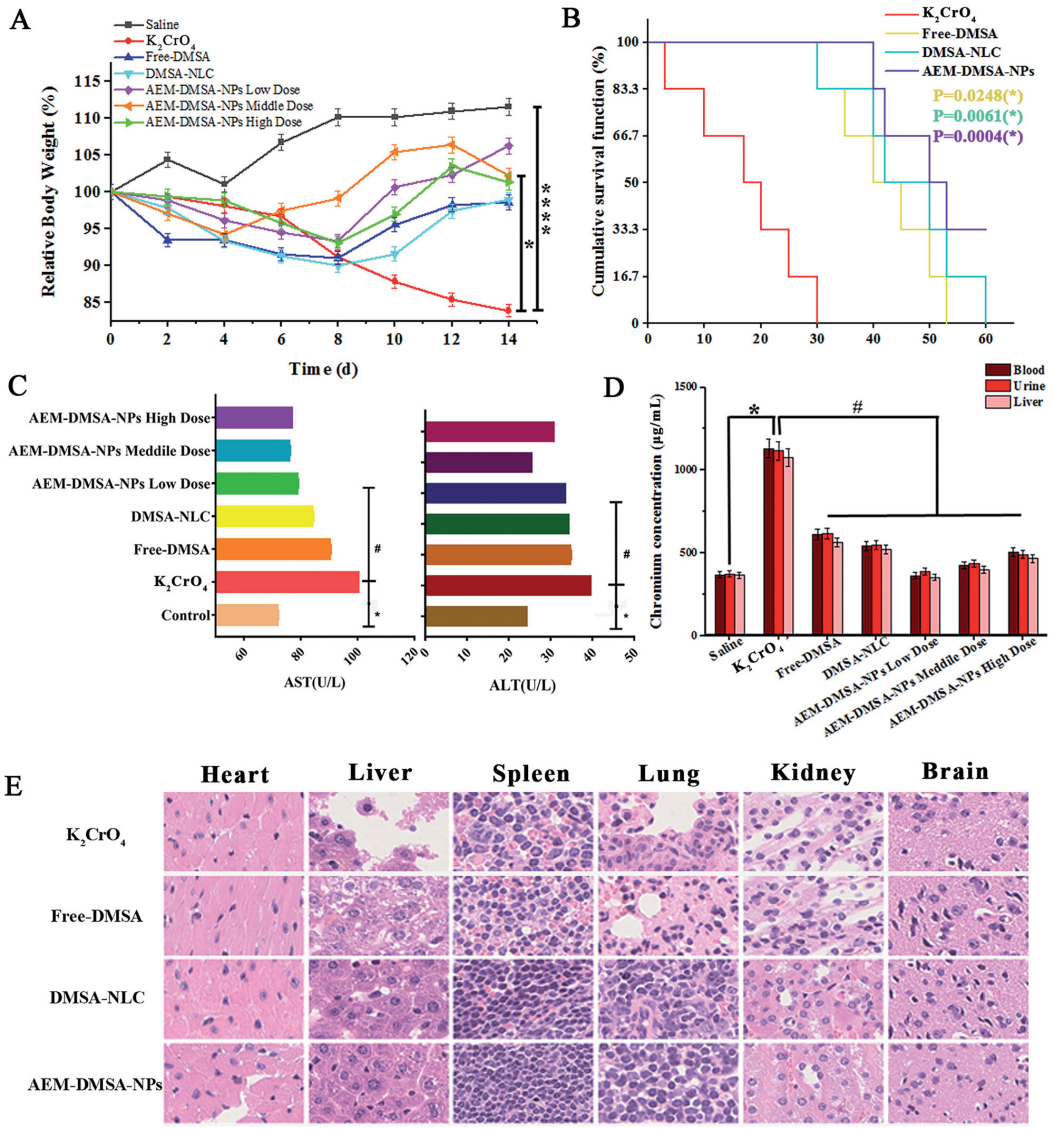
The chromium poisoning detoxifying ability of NPs. (A) Bodyweight changes. The data are presented as the means ± SD (*n* = 3). *indicates *p* < .05. (B) Kaplan–Meier survival curves of KM mice following different treatments. The data are presented as the means ± SD (*n* = 6). (C) ALT and AST in the blood samples from rats with different treatments. (D) Chromium in blood, urine, and liver after different treatments. (E) HE staining of major organ sections after various treatments. The data are presented as the means ± SD (*n* = 3). *p < .05, compared to the control group; #*p* < .05, compared to the K_2_CrO_4_ group.

Changes in the bodyweight of the chromium poisoning model mice were monitored during the experiment. As shown in Figure 4(A), at the end of the experimental period, there was a significant difference in the relative change in body weight between the chromium poisoning group and the control group, and there was also a significant difference between the AEM-DMSA-NP group and the chromium poisoning group. To further investigate the detoxification potential of AEM-DMSA-NPs *in vivo*, a Kaplan–Mayer survival curve was established using KM mice, as shown in Figure 4(B). The AEM-DMSA-NP treatment group showed a significantly extended median survival (50 days) of 2.94-, 1.25-, and 1.19-fold compared to the K_2_CrO_4_ intoxication, free-DMSA, and DMSA-NLC treatment groups, respectively. Both the free-DMSA group (compared to the K_2_CrO_4_ group) (*p* = .0248) and the DMSA-NLC group (*p* = .0061) demonstrated prolonged mean survival in mice. However, the *p*-value of the AEM-DMSA-NP group (*p* = .0004) showed a significantly improved survival rate compared to the other two groups. These trends were consistent with chromium poisoning detoxification.

To further confirm the detoxification and hepatoprotective effects of AEM-DMSA-NPs, we measured alanine aminotransferase (ALT) and aspartate aminotransferase (AST) activity in blood, which are commonly used to evaluate liver function. The results showed (Figure 4(C)) that the liver enzyme activity in the nanomedicine treatment group was significantly decreased compared to the chromium-poisoning group, strongly supporting that the AEM-DMSA-NP medium-dose group could effectively protect liver cells from chromium-induced cell damage.

We further tested the potential use of AEM-DMSA-NPs in a chromium poisoning model. The chromium content in the blood, urine, and liver of mice was analyzed, and the results are shown in Figure 4(D). The chromium content in the blood, urine, and liver of the chromium poisoning group was significantly higher than that of the saline group, and the chromium content in the mice of the DMSA treatment group was significantly decreased, but there was no significant difference between the two groups. The chromium content in the drug treatment group (compared to the K_2_CrO_4_ intoxication group) was significantly different between the drug administration groups. The results showed that AEM-DMSA-NPs had significant pharmacodynamics for the targeted detoxification of chromium poisoning.

Mice were intravenously injected with K_2_CrO_4_, and the treatment was started on the 8th day. After 14 days, HE staining of the sections of the main organs of the mice was observed, as shown in Figure 4(E). The liver and lungs of the K_2_CrO_4_ poisoning group were damaged to a certain extent. The liver, spleen, and lung tissues of the free-DMSA, DMSA-NLC, and AEM-DMSA-NP groups (compared to the K_2_CrO_4_-infected group) were not significantly affected. The HE staining of muscle fibers in each heart tissue was uniform, the epicardium was not thickened, some muscle spaces were not significantly enlarged or smaller, myocardial nuclei were round or oval, the transverse striations of muscle fibers were clear, and no eosinophilic lesions were seen in the muscle fibers (Huang et al., 2018). In the renal tissue, the renal capsule was smooth, the boundary between the cortex and medulla was obvious, the glomeruli in the renal cortex were normal and evenly distributed, and no obvious changes were observed in the renal medulla area. The brain tissue of each group was clear, the nerve cells were closely arranged, the nucleus was clear, and the cytoplasmic nucleus was clearly stained. The results showed that DMSA had low toxicity and a good effect on detoxifying chromium poisoning.

### 3.5. Tissue distribution

**Figure 5.**
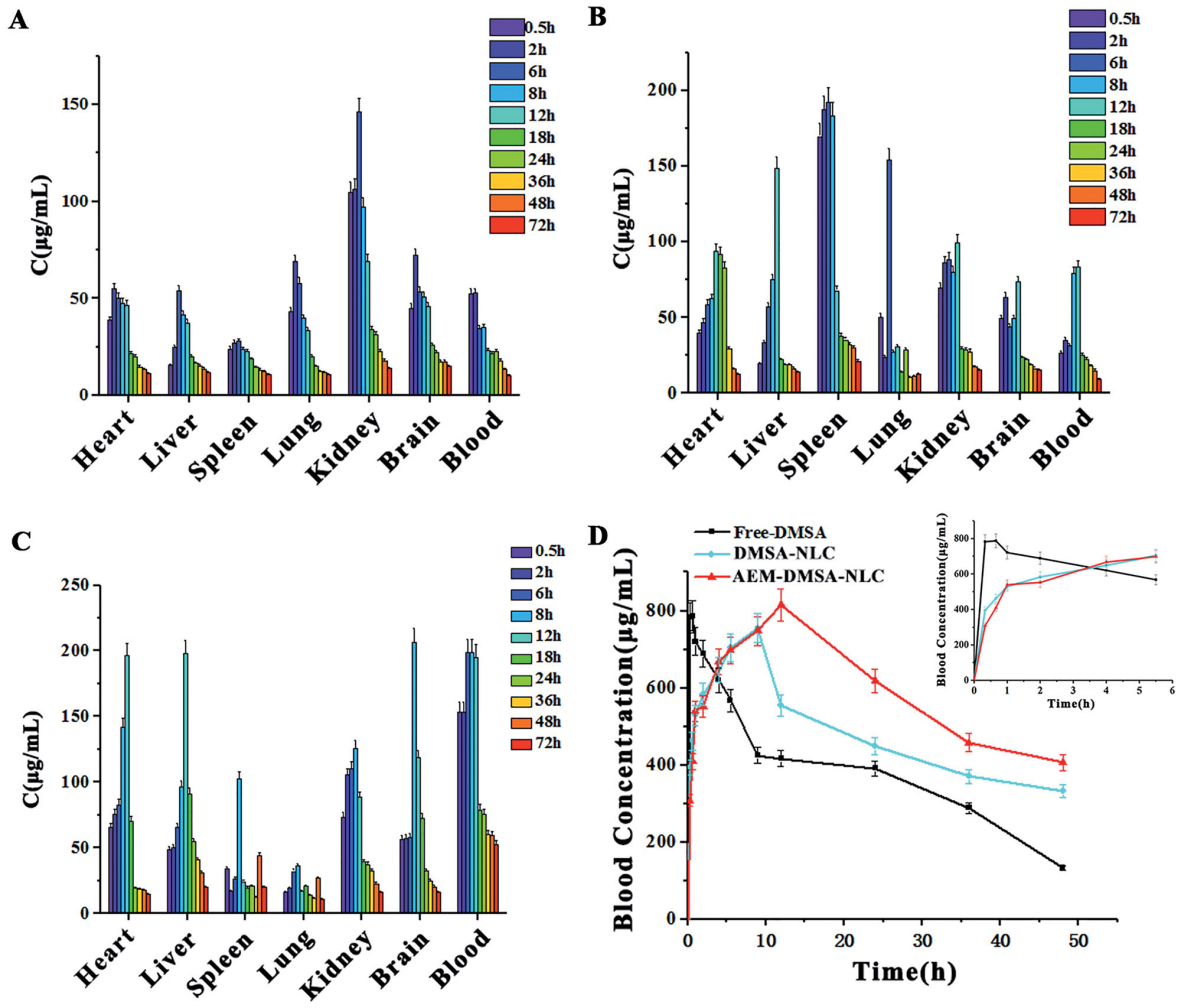
Tissue distribution and pharmacokinetic tests. *In vivo* tissue distribution of free-DMSA (A), DMSA-NLC (B), and AEM-DMSA-NPs (C). (D) Blood concentrations after different treatments. The data are presented as the means ± SD (*n* = 3).

As seen in Figure 5(A), the drug concentrations in the blood, brain, heart, and lung reached a peak 2 h after the administration of free-DMSA, and reached a peak 6 h after administration in other tissues. In each sample, the DMSA concentration from high to low was in the order of kidney, blood, lung, brain, heart, liver, and spleen (Liu et al., 2011). As seen in Figure 5(B), after the intragastric administration of DMSA-NLC to mice, the drug concentrations in the spleen and lung reached a peak 6 h after administration, and the drug concentrations in other tissues reached a peak 12 h after administration. In each sample, the DMSA concentration from high to low was in the order of the spleen, kidney, liver, heart, blood, brain, and lung. As seen in Figure 5(C), after the administration of AEM-DMSA-NPs to mice, the drug concentrations in the heart and liver reached a peak 12 h after administration, and the drug concentrations in other tissues reached a peak 8 h after administration. In each sample, the DMSA concentration from high to low was in the order of blood, liver, kidney, heart, brain, spleen, and lung (Liu et al., 2011). The high content of DMSA in the liver indicated that the drug achieved a liver-targeting effect. Higher concentrations of DMSA were also found in the kidney, indicating that DMSA was mainly metabolized and excreted through the liver and kidneys.

### 3.6. Pharmacokinetic study

At 20 min, 40 min, and 1, 2, 4, 6, 9, 12, 24, 36, and 48 h after caudal vein administration, the blood was collected from the fundus vein cluster of male SD rats, and plasma drug concentrations were analyzed by high-performance liquid chromatography-fluorescence photometry. The curve of the average plasma drug concentration and time was plotted, as shown in Figure 5(D) (He et al., 2014; Li & Feng, 2018). The pharmacokinetic parameters of the AEM-DMSA-NPs, including the AUC_0–24 h_ and clearance (C_L_), were significantly improved.

Pharmacokinetic studies have been used to investigate the *in vivo* behavior of nanomaterials. The blood drug concentration–time data obtained from the experiment were calculated and analyzed by DAS statistical moment model fitting. All data were analyzed by SPSS 11.0 statistical software (SPSS, Chicago, IL) through one-way analysis of variance and *t*-tests. The numerical variable data obtained were expressed as the mean value. *p* < .05, *p* < .01, and *p* < .001 were statistically significant, highly significant, and extremely significant, respectively (Yu et al., 2018). The results of the major pharmacokinetic parameters are shown in Table 1. According to the table, the maximum plasma *C*_max_ of DMSA-NLC and AEM-DMSA-NPs was 755.33 µg/L and 814.72 µg/L, respectively. The *C*_max_ of the DMSA-NLC and AEM-DMSA-NP groups was significantly higher than that of the free-DMSA group. The *T*_1/2_ and *C*_L_ of the DMSA-NLC and AEM-DMSA-NPs were 69.315 and 69.315 h, and 0.237 and 0.233 L/h/kg, respectively, while those of free-DMSA were 19.977 h and 0 L/h/kg, respectively. The results showed that the nanoparticles could slow down the elimination rate of drugs *in vivo* and the clearance rate from plasma, prolonging the action time of the drugs, reducing the dosage of drugs in patients, as well as the side effects of drugs (Yan et al., 2013; Ma, 2015). As shown in Table 1, the pharmacokinetic parameters of the AEM-DMSA-NP group were significantly different from those of the DMSA-NLC group. Based on the above analysis, DMSA modified by aging erythrocyte membranes and made into nanostructured lipid carriers significantly improved the absorption of drugs in the body, and had a higher blood drug concentration, which is more conducive to the role of drugs in treating disease.

**Table 1.** Pharmacokinetic parameters after a single administration of DMSA, DMSA-NLC, and AEM-DMSA-NPs to rats (*n* = 3).

	Free-DMSA	DMSA-NLC	AEM-DMSA-NPs
*T*_1/2_ (h)	19.977	69.315	69.315
*T*_max_ (h)	0.66	9	12
*C*_L_ (L/h/kg)	0	0.237	0.233
*V*_1_ (L/kg)	0.014	23.706	23.319
*C*_max_ (µg/L)	786.96	755.33	814.72
AUC_0–48_ (µg/L*h)	18,141.90	22,917.38	28,280.14
AUC_0–∞_ (µg/L*h)	22,502.42	40,755.44	42,687.70
AUMC_0–48_	340,629.33	473,639.16	604,782.76
MRT_0–48_ (h)	18.776	20.667	21.38
VRT_0–48_ (h^2^)	179.99	198.17	185.36

## 4. Conclusions

In this work, an aging erythrocyte membrane-mediated biomimetic nano-drug delivery system (AEM-DMSA-NPs) was constructed to effectively target the antidote DMSA to the liver for the clinical treatment of chromium poisoning. The biological characteristics of aging erythrocyte membranes provide good biocompatibility for drug-carrying nanosystems circulating *in vivo*, and macrophages facilitate the effective implementation of this strategy for liver targeting. Although preliminary, the data from this work suggest that the biologic properties of aging erythrocyte membranes in combination with appropriate nanoparticles may provide a plausible strategy for biomimetic nano-delivery systems for the hepato-targeted detoxification of chromium poisoning.
